# The Tension-Stiffening Contribution of NSM CFRP to the Behavior of Strengthened RC Beams

**DOI:** 10.3390/ma8074131

**Published:** 2015-07-08

**Authors:** Ahmad Azim Shukri, Kh Mahfuz ud Darain, Mohd Zamin Jumaat

**Affiliations:** 1 Department of Civil Engineering, Faculty of Engineering, University of Malaya, 50603 Kuala Lumpur, Malaysia; E-Mail: ahmadazimshukri@gmail.com; 2 Architecture Discipline, Science, Engineering and Technology School, Khulna University, 9208 Khulna, Bangladesh; E-Mail: khmahfuz@gmail.com

**Keywords:** reinforced concrete, tension stiffening, partial interaction, near-surface mounted (NSM), fiber reinforced polymer (FRP)

## Abstract

Tension stiffening is a characteristic behavior of reinforced concrete (RC) beams which is directly affected by the bond-slip property of steel bar and concrete interfaces. A beam strengthened with a near-surface mounted (NSM) technique would be even more affected by tension stiffening, as the NSM reinforcement also possess a bond-slip property. Yet assessing how much the tension stiffening of NSM contributes to the behavior of RC beams is difficult due to the fact that bond-slip effects cannot be directly incorporated into a strain-based moment-curvature analysis. As such, the tension stiffening is typically incorporated through various empirical formulations, which can require a great deal of testing and calibrations to be done. In this paper a relatively new method, which can be called the mechanics-based segmental approach, is used to directly simulate the tension stiffening effect of NSM reinforcements on RC beams, without the need for empirical formulations to indirectly simulate the tension stiffening. Analysis shows that the tension stiffening of NSM fiber reinforced polymer (FRP) contributes a significant portion to the stiffness and strength of the strengthened RC beam not only during serviceability, but at all load levels.

## 1. Introduction

In recent years, the focus of research on strengthening of reinforced concrete (RC) beams using fiber reinforced polymers (FRP) has changed slightly from externally bonded (EB) techniques [1,2,3] to near-surface mounted (NSM) technique [4,5,6]. NSM FRP has a number of advantages compared to EB FRP in practical applications, such as no surface preparation being required apart from the grooving process and easier accommodation of irregularities on the concrete surface. NSM FRP is also easier to anchor to prevent premature debonding, which makes it particularly attractive, as premature debonding is always a problem in any FRP strengthened structure. Due to being embedded in the concrete, the FRP bars in NSM strengthening has been known to exhibit tension stiffening behavior, prompting several studies on the bond-slip relationship of NSM FRP reinforcements [7,8,9].

Tension stiffening has been known to cause increased flexural rigidity in cracked RC beams. While progress has been made on understanding the tension stiffening of NSM FRP, directly implementing the tension-stiffening effect in simulations had proven difficult. The moment-curvature approach, as a strain-based method, cannot directly incorporate the interface slips that are necessary to simulate tension stiffening. Due of this, empirically derived values or factors are usually introduced to indirectly simulate tension stiffening, such as effective flexural rigidities [10] and hinge lengths [11,12]. These empirical formulations have been noted to be inaccurate when applied to situations outside of the testing regimes that formed them [13].

The limitations imposed by empirically derived values prompted a new analysis approach to be introduced [13,14,15,16,17,18,19]. This new analysis approach aims to simulate the mechanics of RC beams, as seen in practice, such as concrete cracking, crack widening and formation of concrete wedges. This is done through the application of numerical analysis based on the partial interaction theory [14,17,18,19], which enables this new analysis approach to directly incorporate any bond-slip relationship, thereby removing the dependency on the empirical factors to indirectly simulate the mechanics of RC beams as seen in practice. However, it should be noted that while no portion of the mechanics is based on empiricisms, empiricisms are still required in terms of material properties, such as stress-strain relationships and bond-slip relationships.

In this paper, it will be shown how the segmental moment-rotation approach can be used to simulate the behavior of NSM FRP reinforcements. This is done by applying the partial interaction theory to create a numerical analysis that is used to simulate the tension stiffening load-slip relationship between FRP bars, epoxy adhesive and the adjacent concrete area. The moment-rotation approach is then validated against the experimental results by the authors and also against previous published results. The contribution of tension stiffening of NSM FRP reinforcements on the behavior of RC beams at all levels of load up to failure is also verified using a comparison study.

## 2. Segmental Moment-Rotation Approach

**Figure 1:**
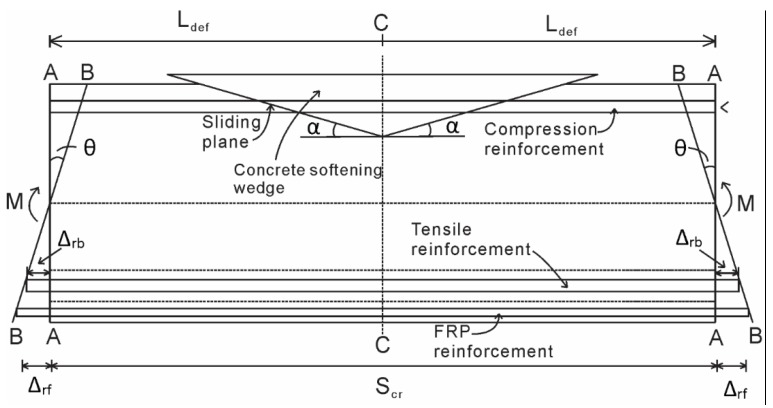
Beam segment of NSM FRP strengthened RC beam.

Consider a NSM FRP strengthened RC beam segment as shown in Figure 1. If a constant moment (*M*) is applied to the segment in Figure 1, the beam ends will rotate (θ) and this rotation is accommodated by an Euler-Bernoulli deformation from section A-A to section B-B, such that plane sections remain planes. The total deformation from *A-A* to *B-B* must be accommodated by a combination of both material strains within the segment and more importantly by the partial-interaction mechanisms.

For NSM FRP strengthened beams, in the tension region, slip between the steel and FRP reinforcement relative to the adjacent concrete will accumulate along the reinforcement, causing the slips Δ_rb_ and Δ_rf_ as shown. In the compression zone, slip across a concrete-concrete sliding plane forms a wedge and accumulates along the sliding plane as shown. The method used to account for these partial-interaction mechanisms will be discussed in the following sections.

### 2.1. Partial-Interaction Tension Stiffening Analysis

The partial interaction theory has been used by several researchers to perform a numerical analysis using bond-slip properties [16,17,18,19], which removes the need for empirically derived formulations for simulating the tension stiffening effect. In this paper it will be shown how the analysis can be adjusted for use in NSM FRP strengthened beams.

**Figure 2:**
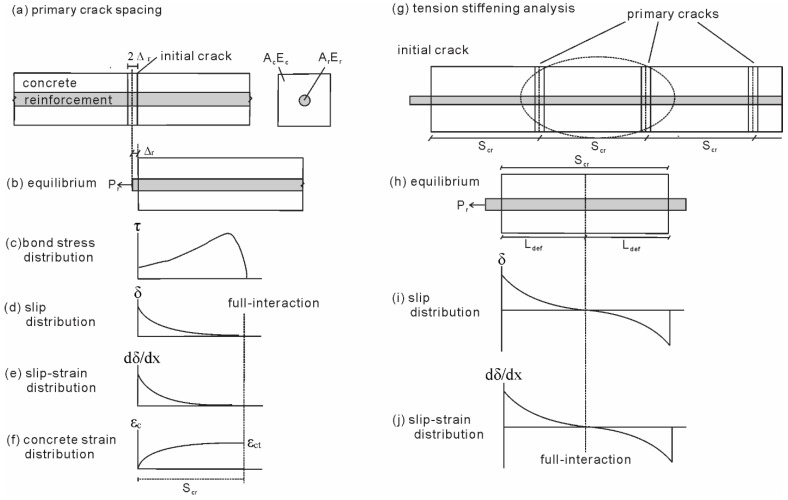
Partial interaction analyses. (**a**) Primary crack spacing; (**b**) Equilibrium; (**c**) Bond stress distribution; (**d**) Slip distribution; (**e**) Slip-strain distribution; (**f**) Concrete strain distribution; (**g**) Tension stiffening analysis; (**h**) Equilibrium; (**i**) Slip distribution; (**j**) Slip-strain distribution.

Prior to cracking of the RC beam, both steel and carbon fiber reinforced polymer (CFRP) reinforcement are in full interaction with adjacent concrete, where due to a perfect bond, both the bars and adjacent concrete are uniformly extended as load is applied to the RC beam. Following the formation of the flexural crack, the steel and CFRP bars are in partial interaction as the bars slip relative to the concrete, resulting in the half crack opening Δ_r_ as shown in Figure 2a,b.

To begin analyzing the effect of slip between the steel and CFRP bars with their adjacent concrete, consider the prism shown in Figure 2. The prism consists of either steel or FRP reinforcement bars of area *A*_r_ and modulus *E*_r_ and the adjacent concrete of area *A*_c_ and modulus *E*_c_. The prism is discretized into very short sections of length d*x*, and at the crack face (the first section) there is no force acting on the concrete as the concrete-concrete interfaces are not touching each other due to the flexural crack. Due to the load *P*_r_, the reinforcement bars slips along the length of the prism as in Figure 2d. This slip is a function of the local bond stress slip (τ/δ) properties. From the distribution of bond stress and knowing the load acting at the crack face, the variation of reinforcement bar and concrete strain can be determined. Hence the slip-strain (dδ/d*x*), which is the difference between the strain in the reinforcement and the concrete, is also known.

Applying the boundary condition that full-interaction is achieved where the interfacial slip and the slip-strain reach zero at the same point, the primary crack spacing *S*_cr_ can be determined as it is known that cracks will form when the strain in the concrete reaches the tensile cracking strain ε_ct_ as shown in Figure 2f [17]. The crack spacing *S*_cr_ represents the minimum crack spacing, as a subsequent crack can form anywhere in the full interaction region to the right of the region *S*_cr_ in Figure 2f. However, if a moment gradient is applied to the beam, which is the usual case, then it is most likely and more conservative to take the primary crack spacing as *S*_cr_ [17].

Once primary cracks have formed along the prism as in Figure 2g, the loading of each prism becomes symmetric. Furthermore, within each primary crack prism, the forces are also symmetric, hence only half the length of a single prism (*L*_def_*)* as in Figure 2h needs to be considered. The load-slip (*P*_r_/Δ_r_) relationship in this case can be determined following the same mechanism as for the infinitely long prism in Figure 2a.

**Figure 3:**
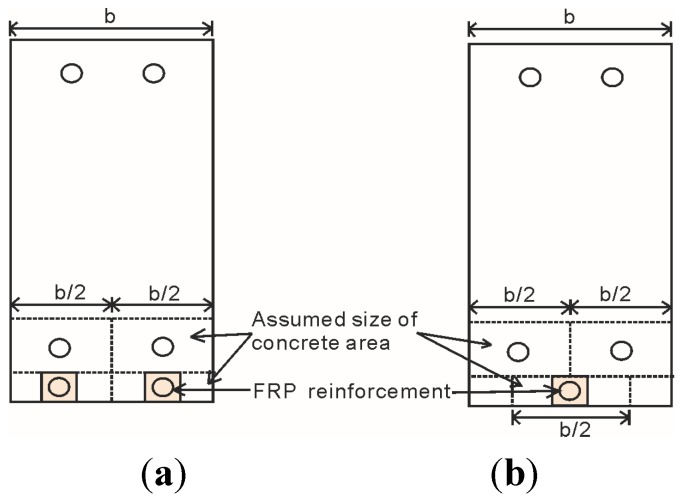
Assumed prism size. (**a**) Concrete size for two FRP reinforcement; (**b**) Concrete size for one FRP reinforcement.

The partial interaction analysis presented above is used to simulate the tension stiffening of steel and also FRP reinforcements between primary cracks. It should be noted that several assumptions were made with respect to NSM FRP strengthening. Firstly, slip is allowed between epoxy-CFRP interfaces, but perfect bonding is assumed between epoxy-concrete interfaces. Secondly, in order to apply the partial interaction theory, the CFRP bar must be located in the center of the prism, such that no moment is induced when the prism is loaded. Hence, it is assumed that the size of the prism for NSM-strengthened beams is as shown in Figure 3, where the smaller concrete area surrounding FRP reinforcement correlates with the lower bond strength of FRP bars compared with steel bars. For the steel reinforcement, the area of concrete should be assumed to be as deep as the bar plus twice the height of the concrete cover, and be as wide as the width of the beam divided evenly between the numbers of steel reinforcement bars. For the FRP reinforcement, when there is more than one FRP reinforcement as in Figure 3a, it is suggested that the depth of the concrete area be taken as the depth of the NSM groove and the width be taken as the width of the beam divided evenly between the numbers of FRP reinforcement bars. When there is only one FRP reinforcement bar, as in Figure 3b, the width is taken as half the beam width.

The partial-interaction analysis is also able to capture the reduced crack spacing that occur due to the application of strengthening as seen in practice. As shown in Figure 3, the application of NSM FRP bar would reduce the available area of concrete around the steel bar. Due to the reduced concrete area, the load transferred from steel bar to concrete would cause a greater strain, meaning the concrete would reach tensile cracking strain faster than it would with a larger available concrete area. This translates to a much smaller primary crack spacing, *S*_cr_.

### 2.2. Size-Dependent Stress-Strain of Concrete

Concrete softening can be captured using any empirically derived stress-strain relationship that takes into account the falling branch of concrete stress-strain. An example of this is the stress-strain model by Popovics [20]. However simply applying the empirical stress-strain relationship to the beam would result in an inaccurate stress-strain curve, as the size of the concrete affects the angle of the concrete wedge that forms during concrete softening [14,15,21], hence affecting the resulting stress-strain curve as well.

**Figure 4:**
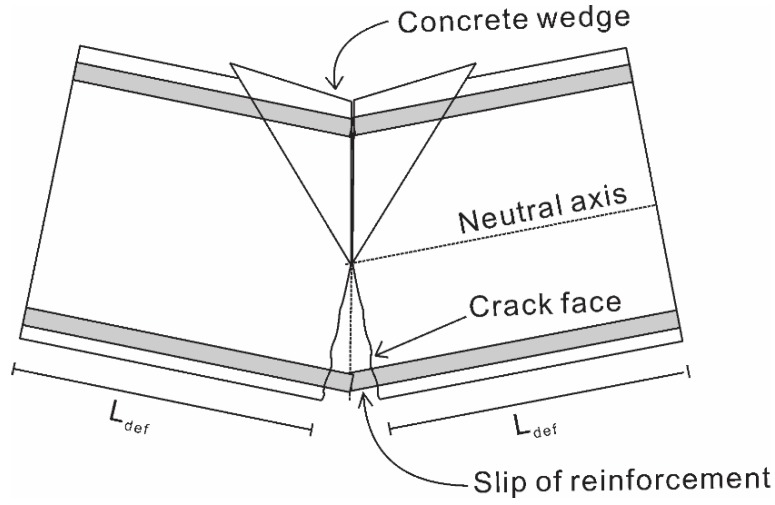
Compression of concrete in hinges.

**Figure 5:**
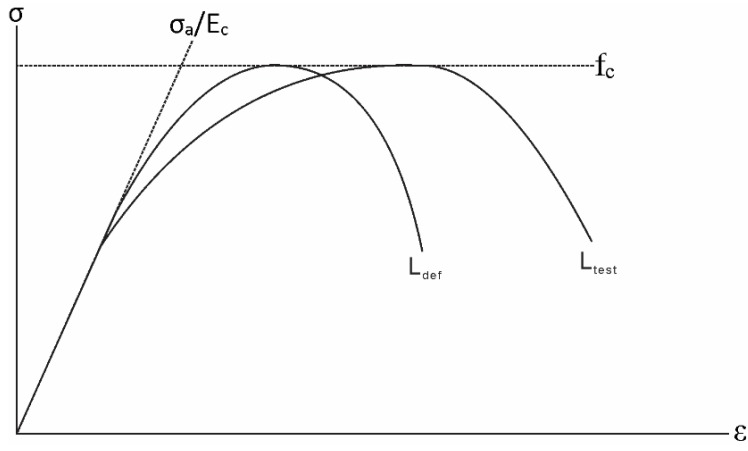
Empirical and size dependent stress-strain curves.

The method proposed by Chen *et al.* [15] can be used to adjust the empirical stress-strain curve. In this method, the height of the concrete cylinders used in forming the empirical stress-strain relationship is designated as 2*L*_test_. The size of the concrete in a beam’s hinge section is taken as 2*L*_def_, as shown in Figure 4. For stress σ_a_/*E*_c_ in the empirical stress-strain relationship for concrete, the strain of concrete that has been adjusted for size is:

(1) $${\text{ε}}_{L_{\text{def}}}=\left({\text{ε}}_{\text{test}}-\frac{{\text{σ}}_{\text{a}}}{E_{\text{c}}}\right)\frac{L_{\text{test}}}{L_{\text{def}}}+\frac{{\text{σ}}_{\text{a}}}{E_{\text{c}}}$$

where ε_test_ is the corresponding strain for stress σ_a_ in the empirical stress-strain relationship and *E*_c_ is the elastic modulus of the concrete. The size-dependent strain should still have the same concrete strength (*f*_c_) while having a different shape compared to the empirical stress-strain relationship, as shown in Figure 5.

### 2.3. Hinge Analysis

**Figure 6:**
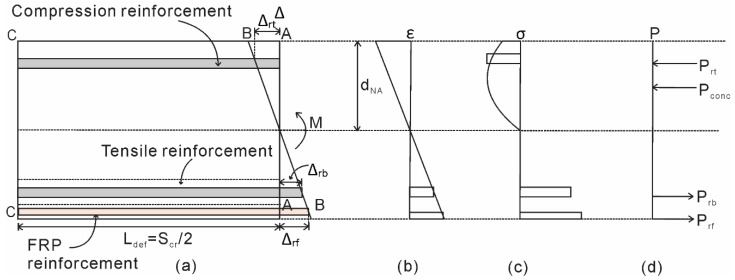
Hinge analysis procedure. (**a**) Beam section; (**b**) Strain profile; (**c**) Stress profile; (**d**) Forces acting on beam section.

The method for obtaining the moment-rotation (*M*/θ) relationship of the beam will now be established. The rotation θ at the end of the hinge in Figure 6a causes an Euler-Bernoulli deformation from A-A to B-B, where plane sections remain plane. In the tension region, a slip between both the steel and NSM reinforcement and the concrete takes place, shown as Δ_rb_ and Δ_NSM_ in Figure 6a. In the compression region, the formation and sliding of concrete wedges takes place.

The rotation causes a deformation profile from A-A to B-B. From this deformation profile, the strain profile (Figure 6b) can be obtained by dividing the deformation profile by *L*_def_. Prior to the formation of flexural cracks, no slip occurs between the concrete and the reinforcement. As such, the stress profile of the beam (Figure 6c) can be determined using the material stress-strain relationship. Note that to determine the forces acting on concrete in the compression region, the size-dependent stress-strain relationship for concrete should be used. The depth of neutral axis d_NA_ is then adjusted until, for a given rotation θ, the equilibrium of forces is achieved, as shown in Figure 6d. The moment, *M*, resulting from the rotation, θ, is then determined. It can be seen that prior to cracking, the procedure is identical to analysis using the moment-curvature approach.

Flexural cracking is considered to have occurred if the rotation causes the strain in the concrete layer around the steel reinforcement to reach the concrete cracking strain. After cracking, the loads developed in the steel bar (*P*_rb_) and the NSM reinforcement (*P*_FRP_) are no longer functions of the strain profile due to the partial interaction that occurs due to the bond with the adjacent concrete. Therefore, the *P*_rb_ and *P*_FRP_, as shown in Figure 6d, are determined by using the deformation profile to obtain the slip values of Δ_rb_ and Δ_FRP_, and using the load-slip relationship obtained using the numerical partial interaction analysis. Note that this requires two separate load-slip relationships, one for the steel reinforcement and the other for the FRP reinforcement. The depth of the neutral axis, d_NA_, is then adjusted until equilibrium of the forces is achieved. The moment is then calculated and the analysis is repeated until the required moment-rotation relationship is obtained. To change the rotation to curvature is simply a matter of dividing the rotation, θ by *L*_def_. Using the moment-curvature relationship, the load-deflection of the beam can be determined using the commonly used double integration method.

## 3. Experimental Program

In order to validate the segmental moment-rotation approach, a simple experimental programme was carried out on NSM strengthened RC specimens. One RC beam was prepared without any strengthening material and was tested as the control specimen. Two RC specimens were strengthened using a NSM CFRP bar with variable bond length (1800 and 1900 mm). To complement this experimental programme, the data for other specimens were also taken from the published results of various researchers [22,23,24]. The testing matrix is shown in Table 1.

**Table 1:** Specimens List.

Specimen name & details	Bonded length (mm)	Beam length (mm)	Shape of NSM CFRP reinforcement	Number of NSM FRP reinforcements
CB (Control beam)	-	2000	-	-
A1 (Strengthened beam)	1800	2000	bar	1
A2 (Strengthened beam)	1900	2000	bar	1
Jung-1 [22]	2700	3000	bar	1
Capozucca-1 [23]	1300	1500	bar	2
Teng-1 [24]	1800	3000	strip	1
Teng-2 [24]	2900	3000	strip	1

### Materials and Samples

**Figure 7:**
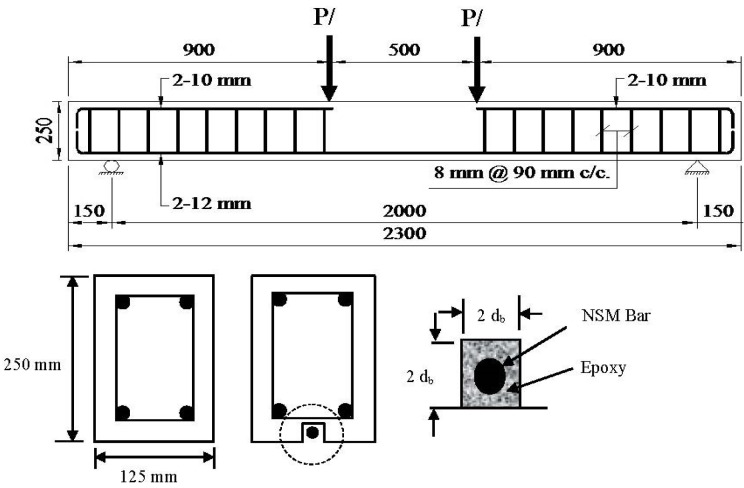
Specimen details.

Rectangular 2.3 m long RC beams with 125 mm × 250 mm cross-sectional dimensions were selected for the experimental study. The dimensions and material properties for the other researchers’ work can be found elsewhere [22,23,24]. The beams were designed to be under-reinforced (ρ = *A*_s_/*bd* = 0.0085, where *b* is beam width and *d* is depth of beam) with two 12 mm diameter deformed bars as tensile reinforcement. Two 10 mm diameter deformed bars were used as hanger bars to hold the stirrups. Double-legged closed 8 mm diameter steel stirrups were used as shear reinforcement with a spacing of 90 mm centre-to-centre to ensure that a flexure failure would occur. Figure 7 exhibits the features of the beam arrangement.

The concrete cube compressive strength, concrete cylinder compressive strength and flexural strength of the concrete were determined according to BS EN 12390-3:22009, ASTM C39 and BS EN 12390-5:22009 [25,26,27] respectively. The material properties of concrete are described in Table 2. The mechanical properties of the deformed steel bars supplied by the manufacturer were checked in the laboratory to determine whether they conformed to the ASTM A615 [28] specification. Lamaco System (Alor Setar, Malaysia) supplied the 12 mm diameter sand coated carbon-epoxy pultruded FRP (CFRP) bars with a density of 1.65 g/mm^3^ for NSM strengthening. These CFRP bars showed linear elastic response up to ultimate failure. To adhere the NSM bar to the concrete substrate, Sikadur^®^ 30 (Sika Malaysia, Kuala Lumpur, Malaysia) was used as epoxy adhesive. The details of the concrete, steel and CFRP material properties are described in Table 2 and the Sikadur^®^ 30 properties [29] at temperatures of 15 °C and 35 °C are mentioned in Table 3. The values provided in Table 2 are the average values from three samples.

**Figure 8:**
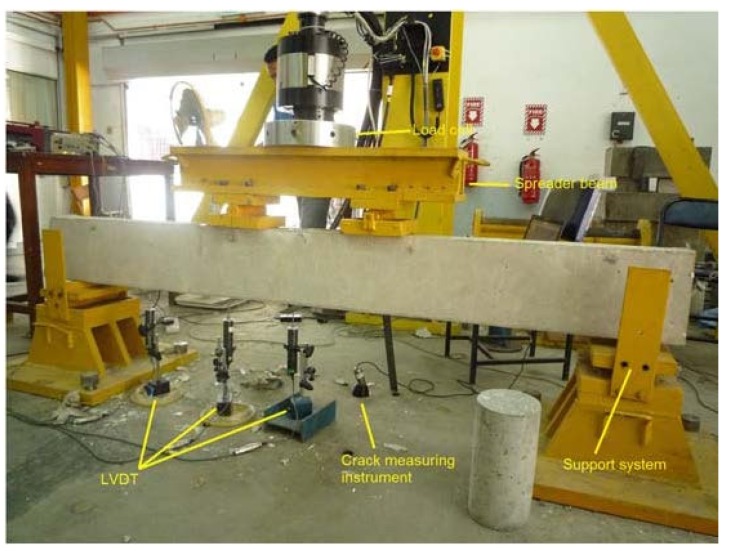
Experimental setup.

Each of the strengthened beam specimens had a single groove (24 mm × 24 mm) made using a diamond-bladed concrete saw along the beam length to accommodate the CFRP bar. A hammer and hand chisel were used to remove the remaining concrete lugs from the groove and all the debris was then removed from the groove using airbrushing pressure. An epoxy adhesive (Sikadur^®^ 30) was applied into the groove to fill around two-thirds of the groove depth. The CFRP bar was then gently inserted into the groove, and pressed lightly to ensure proper epoxy covering surrounding the bar. The groove’s outer surface was then levelled and left for one week to achieve proper epoxy strength. The beams were then tested under static loading using the four-point loading test. The setup of the four-point loading test is shown in Figure 8.

**Table 2:** Properties of Concrete, Internal Steel Reinforcement and CFRP Bar. Φ = diameter.

Material	Compressive strength (MPa)	Flexure strength (MPa)	Yield stress (MPa)	Ultimate strength (MPa)	Elastic modulus (GPa)	Strain at failure (%)
Concrete	43.24 (cube) 35.63 (Cylinder)	5.01	-	-	30.1	-
Steel- Φ 12 mm	-	-	520	587	200	20
Steel- Φ 10 mm	-	-	529	578	200	21
Steel- Φ 8 mm	-	-	380	450	200	29
CFRP- Φ 12 mm	-	-	-	2400	165	-

**Table 3:** Properties of Sikadur^®^ 30 [29].

Temperature	15 °C	35 °C
Compressive strength	70–80 MPa	85–95 MPa
Tensile strength	14–17 MPa	16–19 MPa
Shear strength	24–27 MPa	26–31 MPa

## 4. Results and discussion

### 4.1. Experimental Results

**Figure 9:**
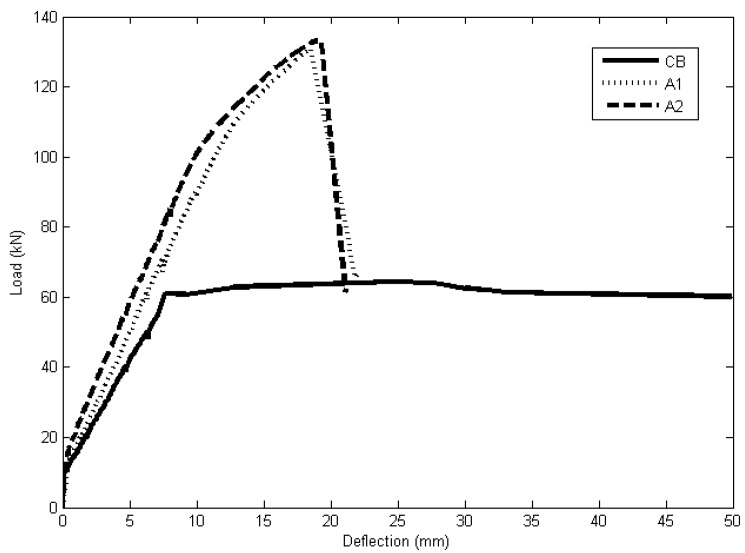
Load-deflection result for beams CB, A1 and A2.

The load *versus* mid-span deflection curves for the beams tested by the authors are shown in Figure 9. The curves can be approximately divided into three distinct phases. The first segment of the curve shows the pre-cracking phase, where the curve linearly varies with very negligible deflection up to the occurrence of first flexural cracking. Strengthening using NSM CFRP was found to increase the load level at which the first flexural crack occurred for all the strengthened beams.

**Figure 10:**
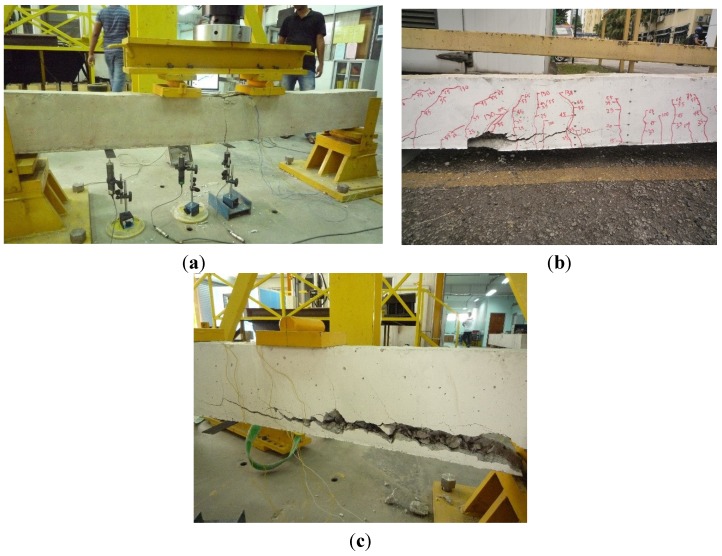
Failure modes of beam CB, A1 and A2. (**a**) Beam CB (concrete crushing failure); (**b**) Beam A1 (concrete cover separation); (**c**) Beam A2 (concrete cover separation).

The second phase is from the first flexural cracking to yielding of the internal reinforcement of the beams, where a loss of stiffness can be observed. The strengthened beams were found to show higher stiffness compared to the control beam due to the CFRP bars in this phase. The third portion of the load-deflection behaviour is from yielding until failure. The strengthened beams show improved strength and stiffness compared to the control beam. While the control beam failed by concrete crushing after steel yielding, both the strengthened beams failed by concrete cover separation. A summary of the experimental test results is given in Table 4, while the failure modes of the beams are shown in Figure 10.

**Table 4:** Summary of Experimental Test Results. *P*_cr_ = first crack load; *P*_y_ = yield load; *P*_u_ = ultimate load; ∆_y_ = deflection at yield of steel; ∆_u_ = mid-span deflection at failure load.

Specimen name	*P*_cr_ (kN)	*P*_y_ (kN)	Δ_y_ (mm)	*P*_u_ (kN)	Δ_u_ (mm)	Failure mode
CB	10.6	61.0	7.7	64.4	24.7	Flexure failure (Concrete crushing after steel yielding)
*A*1	14.0	110.6	13.0	130.8	18.6	Debonding (Concrete cover separation)
*A*2	15.0	104.5	10.7	133.2	19.2	Debonding (Concrete cover separation)

### 4.2. Validation of Segmental Moment-Rotation Approach

The experimental results from the authors, and also from previously published works, were simulated using the segmental moment-rotation approach. Several generic material models were used to obtain the simulated results. For the partial interaction analysis, the material models used are the local bond strength for FRP bar by Hassan and Rizkalla [30], local bond strength for FRP strip by Zhang *et al.* [31], bond stress-slip relationship for FRP bar by Lorenzis [32], and bond stress-slip relationship for FRP strip by Zhang *et al.* [31]. For concrete softening, the size dependent stress-strain is obtained from the empirical concrete stress-strain relationship by Popovics [20]. As the segmental moment-rotation approach is generic, any other material model can be used if it is believed that it can provide a better result compared to the ones used in this paper.

**Figure 11:**
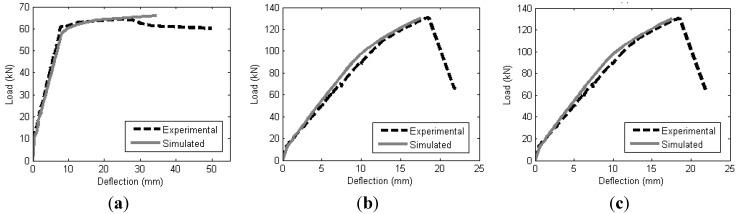
Comparison of experimental and simulated load-deflection of beams CB, A1 and A2. (**a**) CB; (**b**) A1; (**c**) A2.

**Figure 12:**
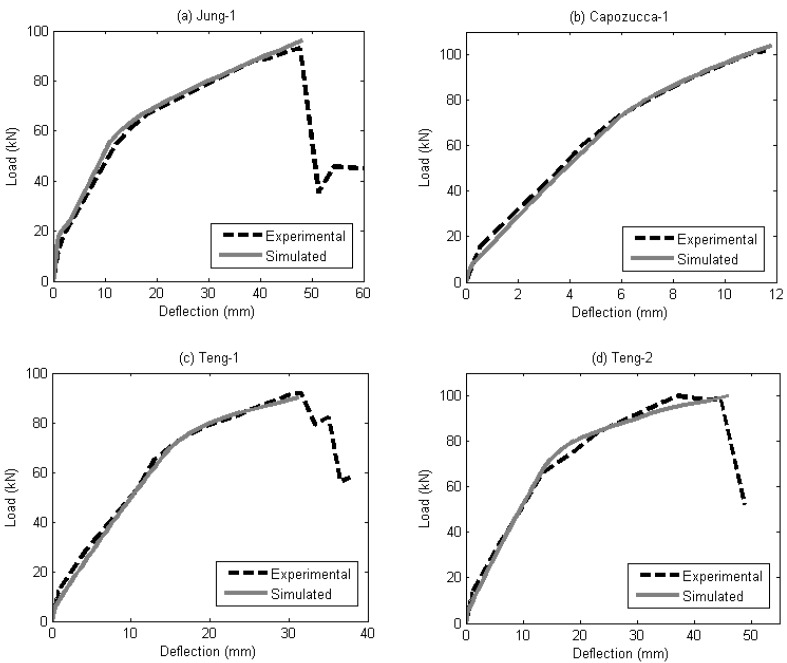
Comparison of experimental and simulated load-deflection of beams (**a**) Jung-1; (**b**) Capozucca-1; (**c**) Teng-1; and (**d**) Teng-2.

Comparisons between simulated and experimental load-deflection are shown in Figure 11 and Figure 12. It can be seen that the analysis provides good agreement with the experimental results as the peak points and the shape of the load-deflection curves were captured reasonably well. The analysis is also able to capture the effect of the different CFRP-bonded lengths of the beam specimens and is proven to work on both bar-shaped CFRP reinforcement and strip CFRP reinforcement. Currently, the segmental approach presented in this paper is unable to predict premature debonding failures, such as the concrete cover separation failure mode.

**Figure 13:**
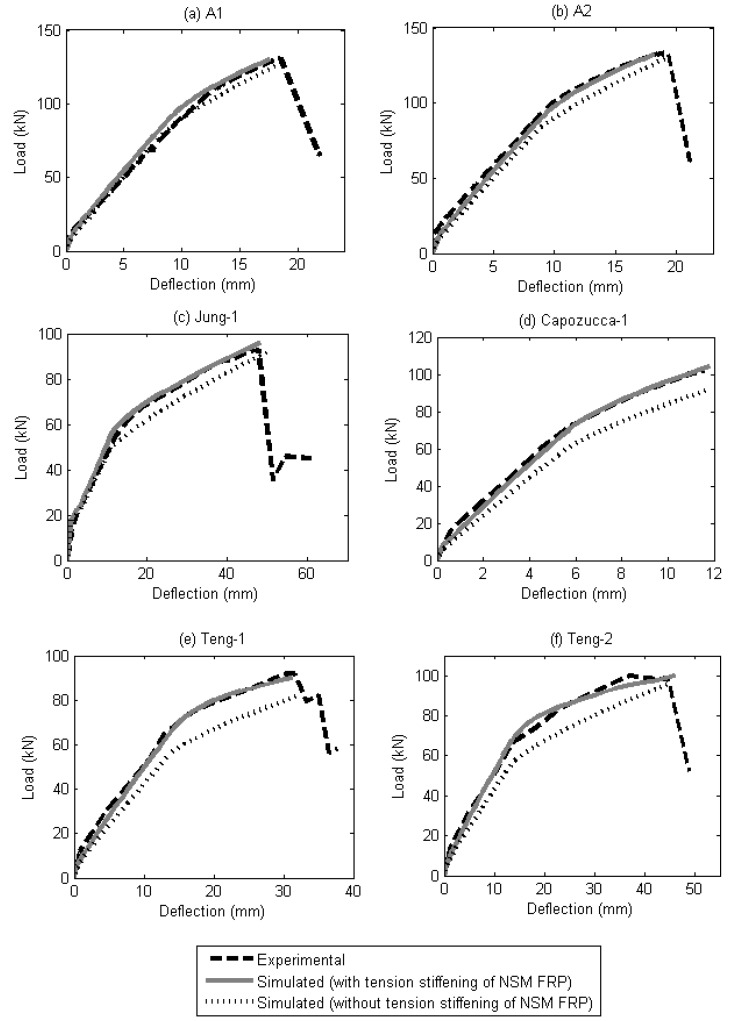
Effect of tension stiffening of NSM reinforcement. (**a**) A1; (**b**) A2; (**c**) Jung-1; (**d**) Capozucca-1; (**e**) Teng-1; (**f**) Teng-2.

To determine the contribution of the tension-stiffening effect of NSM FRP reinforcements to the behaviour of RC beams, a comparison is made in Figure 13. It is emphasized here that the tension-stiffening effect that is simulated using the partial interaction analysis in this study refers to the tension stiffening seen at all levels of load up to failure, rather than simply at the serviceability load level. From Figure 13, it can also be seen that the tension-stiffening effect in beams strengthened using NSM CFRP strips (Teng-1, Teng-2) is greater than in beams strengthened using NSM CFRP bars (A1, A2, Jung-1 Capozucca-1). This can be attributed to the better bonding that NSM CFRP strips generally has compared to NSM CFRP bars [33].

## 5. Conclusions

In this study, the segmental moment-rotation approach was used to predict the load-deflection behaviour of NSM FRP-strengthened beams. Through the application of partial interaction theory, the bond properties of the NSM FRP can be directly simulated, thereby allowing the tension-stiffening behaviour to be simulated for all level of loads of the strengthened RC beam. The model is generic, allowing it to cope with any type of bond and material properties for the NSM FRP reinforcement used. The results from the model have shown to be in good agreement with experimental results. It was also shown that the tension-stiffening behaviour of NSM FRP plays an important role in the behaviour of strengthened RC beams for all load levels.
